# Prevalence and associated factors of the career plateau of primary care providers in Heilongjiang, China: a cross-sectional study

**DOI:** 10.1186/s12875-021-01389-w

**Published:** 2021-02-17

**Authors:** Di Liu, Xu Yang, Qinglin Li, Lei Shi, Qiaoran Tang

**Affiliations:** 1 School of Economics and Management, Harbin Engineering University, Harbin, People’s Republic of China; 2 School of Marxism, Harbin Medical University, Harbin, People’s Republic of China; 3 School of Health Management, Harbin Medical University, Harbin, People’s Republic of China; 4 Children’s Hospital of Soochow University, Suzhou, People’s Republic of China

**Keywords:** Primary care providers, Career plateau, Related factors, Path to improvement

## Abstract

**Background:**

Primary care providers are pillars of China’s medical and health sectors. However, due to the gap between career expectations and reality, they enter a career plateau phase through excessive pressure. This study aims to examine the prevalence and associated factors of the career plateau of primary care providers in Heilongjiang Province, China, and proposes strategies to improve and promote their career advancement.

**Methods:**

Based on city-level GDP growth in the province, a questionnaire survey was conducted among 1500 primary care providers (effective response rate = 85.8%). Pearson’s chi-square analysis and binary logistic regression were used to analyse the factors associated with their career plateau.

**Results:**

The prevalence rate of career plateau was 61.8% among primary care provider respondents. The factors associated with a career plateau included having a spouse (OR = 1.394, 95%CI = 1.056–1.839), working more than 40 h per week (OR = 1.473, 95%CI = 1.146–1.893); working for 11–20 years (OR = 1.607, 95%CI = 1.150–2.246); working for more than 20 years (OR = 2.818, 95%CI = 1.938–4.097); having an average monthly income of 3001–4000 yuan (OR = 1.886, 95%CI = 1.197–2.969) or 4001–5000 yuan (OR = 2.104, 95%CI = 1.256–3.524); and reporting unsatisfactory or very unsatisfactory sleep quality (OR = 1.838, 95%CI = 1.317–2.567).

**Conclusions:**

Primary care providers in Heilongjiang Province face a high career plateau, with marital status, weekly working hours, number of years employed, monthly average income, and sleep quality considered associated factors. To eliminate negative factors of the career plateau, it is necessary to provide support to primary care providers in four domains: individual, organisation, society, and policy.

**Supplementary Information:**

The online version contains supplementary material available at 10.1186/s12875-021-01389-w.

## Background

With a rapidly aging world population and increases in disease risk factors, the existing medical and health resources are increasingly less able to meet increasing demand for health services [1]. As providers of primary health services, community-level health service personnel assume multiple responsibilities of prevention, health care, rehabilitation, health education, family planning technical services, and the diagnosis and treatment of common and frequently occurring diseases. They play an important role in the medical and health systems of various countries. Focussing on the career development of grassroots health service personnel is conducive to the improvement of their service capacity and alleviation of the contradiction between medical supply and demand [2]. In recent research on the careers of primary care providers [3–6], scholars have mainly focussed on their high sense of burnout; however, the attention paid to their career development status, that is, career plateau, is clearly insufficient.

The concept of a career plateau was first proposed by American psychologist Ference in 1977 and can be defined as a state in which individuals have a limited possibility of future growth in their careers [7]. The career plateau marks stagnation in both the vertical and horizontal flow of jobs [8], where the possibility of individuals undertaking further responsibilities and challenges in their careers is negligible [9, 10]. In previous studies, researchers have provided various classifications of career plateaus [7, 11–16]. Considering the cultural adaptability of China, in this study, career plateaus can be categorised as hierarchical, content, and central plateaus. The factors associated with career plateaus are complex and varied. Feldman et al. [9] believe that the career plateau is associated with six factors: individual ability and technology, individual needs and values, pressure, internal motivation, external reward, and organisational growth. Tremblay et al. [17] indicated that the career plateau of employees is primarily associated with three major factors: individual, family, and organisation. Meanwhile, Zhang et al. [18] indicated that factors such as top-level design, institutional management, and individual attribution were responsible for the career plateau of workers. In most studies [19–22], it is stated that gender, professional title, educational background, age, working years, working time, and marital status are related factors of career plateau. It is believed that the emergence of a career plateau is related to work performance, organisational commitment, turnover intention, and job satisfaction among employees [23–25], not only triggering psychological and physiological health issues in employees but also reducing organisational management efficiency and work performance, resulting in a loss of organisational talent [26, 27].

Research on the career plateau of medical and health workers [21, 22, 28–30] has mainly focussed on nurses, doctors, and other groups, with little attention to primary care providers. Only a few Chinese scholars have carried out research on such care providers. According to the research of Zhang et al. [18], the career plateau of primary care providers is particularly noticeable. In China, primary care providers (referring to health care providers in urban and rural basic medical and health institutions, specifically urban community health service centres/stands in towns and townships and rural health centres/institutions) are the mainstay of the medical and health fields, supporting the heavy medical task and contributing significantly towards China’s health and economic development. In the prevention and control of COVID 19 virus, four million primary care providers in China were observed to be fighting the disease on the front line. They not only face such problems as scarce medical resources and difficult working conditions to secure career development [18, 31], but also situations where leading hospitals siphon patients, causing a shortage of patients. Moreover, China’s primary-level health service institutions are short of funds and resources, with minimal external repair opportunities and insufficient talent training [18]. Further, in terms of institutional management, there is a lack of mechanisms and management [18]. In Chinese society, there is poor recognition of primary care providers [31], inadequate social respect and support, and strained doctor-patient relationships [32]. Under the new medical reform policy, the contract service of family doctors lacks incentive measures for primary care providers [3], and the extra burden without additional pay is bound to affect their enthusiasm and sense of gain [31]. Faced with the gap between career expectations and reality, primary care providers enter a career plateau phase due to excessive pressure [32].

A career plateau hinders career advancement and affects the ability to serve the public and ensure the satisfaction of patients and their health outcomes [18]. It is imperative to consider the career plateau of primary care providers to better understand their working status, avoid the loss of basic medical talents, ease the gap between the shortage of medical supply and demand, and better serve the Healthy China 2030 Strategy [18, 19]. However, at present, only a few researchers in China have focused on the incidence of career plateau among primary care providers. This study aims to fill this gap and examine the career plateau prevalence rate and associated factors among primary care providers. The knowledge obtained from the study can be used to promote their career development, and provide a theoretical reference for the sound development of basic health care.

## Methods

### Participants and procedures

The questionnaire survey was conducted in Heilongjiang Province, China, from June to August 2019. According to the GDP of prefecture-level cities in the province, as well as the provincial statistical yearbook for 2019 [24], there are 18,460 basic medical and health institutions in Heilongjiang, including 614 county/district community health service stations, 976 township hospitals, 10,740 village clinics, and 6130 outpatient clinics. Among them, 15,007 were community primary care providers. Through stratified sampling, 1500 primary care providers from 38 community health service stations/centres in seven cities in the province were selected as study subjects. Before commencing the survey, the researchers communicated and coordinated with the research community health service centres, and after obtaining their consent, the questionnaire was distributed to the community health service centres. Among the 1348 questionnaires collected, those with obvious errors, incorrect answers to polygraph questions, and incomplete answers were eliminated, and a total of 1287 valid questionnaires were obtained, with an effective recovery rate of 85.8%.

Inclusion criteria: staff who have been engaged in basic medical and health institutions for one year or more; and informed consent to participate in this study. Exclusion criteria: those who have been on the job for three months or more due to accumulated study, study or sick leave within one year; and security staff at primary health centres, interns, and training staff.

### Questionnaire

A self-made general demographic questionnaire and the career plateau scale compiled by Baoguo Xie were employed in this study, and before the formal investigation, 50 primary care providers were selected in Harbin for a preliminary survey. According to the preliminary survey results and expert opinions, the questionnaire items of the scale were partially modified to form the final questionnaire.

### Demographic characteristics

The study adopted a self-compiled general demographic questionnaire, which comprised 10 items— gender, marital status, age, educational background, work years, monthly average income, work hours per week, flexible job system, type of personnel post allocation, and sleep quality [See Additional file 1].

### Career plateau scale

The career plateau scale compiled by the Chinese scholar Baoguo Xie [12] was adopted to measure the career plateau, with a total of 16 items, including 4 positive and 12 negative items. The scale included three dimensions—central plateau (six items), content plateau (six item s), and hierarchical plateau (four items)—that have been widely used in the medical and health fields in China (such as doctors, nurses, and health technicians) [23, 24, 33, 34]. A six-point Likert scale was adopted for the scale, and the positive items were assigned values according to the following: 1 = *totally disagree*, 2 = *relatively disagree*, 3 = *somewhat disagree*, 4 = *somewhat agree*, 5 = *relatively agree*, 6 = *fully agree*; the negative items were reverse-scored when calculating the average score of the career plateau and each of the three dimensions. The higher the score, the greater the possibility of entering the career plateau. An average score was equal to or greater than 4 points was considered indicative of a career plateau. The Cronbach’s alpha for this scale was 0.835, and its KMO coefficient was 0.943 [See Additional file 1].

### Data analysis

The study used IBM SPSS 20.0 for statistical analysis, with a career plateau score of 4 considered the threshold: Those scoring greater than or equal to 4 were classified as the career plateau group, and those less than 4 as the non-career plateau group. Adopting a descriptive statistical analysis method, the authors analysed and described the survey subjects’ career plateau levels and the mean and standard deviation of each dimension. The chi-square test was used to compare the incidence of career plateau with different demographic information. Career plateau was the dependent variable, and gender, marital status, type of establishment, weekly working hours, number of years on the job, monthly average income, and sleep quality were treated as independent variables in multivariate binary logistic regression with 95% confidence intervals for both tests to analyse the factors associated with the career plateau. *P* < 0.05 was considered statistically significant.

## Results

### Demographic characteristics of the respondents

The involvement in the research of primary care providers accorded priority to women (83.6%), those aged 31–40 years (34.9%), having a spouse (68.5%), education with college degree or above (53.3%), 40 h a week working time more or less (64.5%), working for a fixed number of years—10 years or less (54.7%), non-flexible job profiles (73.0%), types for appointment system (37.5%), average monthly income of 2000–3000 yuan (34.2%), and quality of sleep (41.3%). See Table 1 for details.

**Table 1:** career plateau chi-square of survey subjects (*N* = 1287)

Demographic characteristics	N	%	The number of people on the career plateau	Percentage of career plateaus	χ^2^	P
Gender						
Male	211	16.4	140	66.4	2.167	0.141
Female	1076	83.6	656	61.0		
Age group						
≦30 years old	427	33.2	158	37.0	177.70	< 0.001
31–40 years old	449	34.9	315	70.2		
41–50 years old	327	25.4	249	76.1		
≧51 years old	84	6.5	74	88.1		
Marriage status						
Have a spouse	882	68.5	589	66.8	28.879	< 0.001
No spouse	405	31.5	207	51.1		
Record of formal schooling						
High school degree or below	173	13.4	123	71.1	7.300	0.026
University college	429	33.3	261	60.8		
University degree or above	685	53.3	412	60.1		
Weekly working hours						
≦ 40 h	830	64.5	495	59.6	4.841	0.028
> 40 h	457	35.5	301	65.9		
Working fixed number of year						
≦ 10 years	704	54.7	372	52.8	59.385	< 0.001
11–20 years	256	19.9	172	67.2		
≧20 years	327	25.4	252	77.1		
Flexible job system						
Yes	348	27.0	215	61.8	0.001	0.976
No	939	73.0	581	61.9		
Type of personnel post allocation						
Utilities staffing	481	37.4	319	66.3	7.963	0.019
Appointment system	483	37.5	294	60.9		
Equal pay for equal work /Temporary recruit	323	25.1	183	56.7		
Average monthly income						
<2000 yuan	127	9.9	57	44.9	32.544	< 0.001
2001–3000 yuan	440	34.2	252	57.3		
3001–4000 yuan	331	25.7	213	64.4		
4001–5000 yuan	215	16.7	153	71.2		
>5000 yuan	174	13.5	121	69.5		
Quality of sleep						
Very satisfied and relatively satisfied	228	17.7	126	55.3	19.452	< 0.001
General	531	41.3	306	57.6		
Relatively dissatisfied and very dissatisfied	528	41.0	364	68.9		

### Descriptive analysis of the score of career plateau

The results showed that primary health care workers were in a career plateau state overall, with an overall plateau score of 4.07 ± 0.77. The centralised plateau score was the highest (4.48 ± 1.33), followed by the work content plateau (4.46 ± 0.85), while the hierarchical plateau score was the lowest (2.97 ± 0.92).

### Associations between different demographic variables and career plateau

The chi-square test results showed that the career plateau prevalence rate of primary care providers was 61.8% (796/1287). Compared to the non-career plateau group, age (χ^2^ = 177.70, *p* < 0.001), education (χ^2^ = 7.3, *p* = 0.026), marital status (χ^2^ = 28.88, *p* < 0.001), weekly working hours (χ^2^ = 4.841, *p* = 0.028), number of years on the job (χ^2^ = 59.385, *p* < 0.001), sleep quality (χ^2^ = 19.452, *p* < 0.001), monthly income (χ^2^ = 32.544, *p* < 0.001), and other aspects of the career plateau group were significantly different. See Table 1 for details.

### Binary logistic regression analysis results

The results of binary logistic regression (Table 2) showed that marital status, weekly working hours, number of years on the job, monthly average income, and sleep quality were the factors associated with the career plateau of primary care providers. In terms of marital status, those with a spouse had a higher chance of hitting a career plateau (OR = 1.394, *p* < 0.05) than primary care providers at the basic level without a spouse. There was a clear positive correlation between weekly working hours of over 40 h (OR = 1.473, *p* < 0.05) and the career plateau; that is, the longer the weekly working hours of primary care providers, the higher the incidence of the career plateau. In terms of the number of years on the job, compared with primary care providers whose working years were ≤ 10 years, the career plateau of those with working years ranging from 11 to 20 years (OR = 1.607, *p* < 0.05) and ≥ 20 years (OR = 2.818, *p* < 0.05) registered a higher probability. With respect to monthly average income, compared with primary care providers whose monthly average income was less than 2000 yuan, the incidence of career plateau for those with a monthly average income of 3001–4000 yuan (OR = 1.886, *p* < 0.05) and 4001–5000 yuan (OR = 2.104, *p* < 0.05) was higher. In terms of sleep quality, the possibility of a career plateau for primary care providers with unsatisfactory and very unsatisfactory sleep quality (OR = 1.838, *p* < 0.05) was higher than for those with satisfactory and relatively satisfactory sleep quality. See Table 2 for details.

**Table 2:** binary logistic regression analysis

Statistical variables	The assignment case	Regression coefficient	Standard error	Wals	P	OR	95%CI
Gender	1 = Male	0.184	0.167	1.215	0.27	1.202	0.867–1.667
	2 = Female					contrast	
Marriage status	1 = Have a spouse	0.332	0.141	5.507	0.019	1.394	1.056–1.839
	2 = No spouse					contrast	
Weekly working hours	1 = ≦40 h					contrast	
	2 = >40 h	0.387	0.128	9.166	0.002	1.473	1.146–1.893
Working fixed number of year	1 = ≦10 years					contrast	
	2 = 11–20 years	0.474	0.171	7.707	0.006	1.607	1.150–2.246
	3 = ≧20 years	1.036	0.191	29.45	< 0.001	2.818	1.938–4.097
Type of personnel post allocation	1 = Utilities staffing					contrast	
	2 = Appointment system	0.323	0.171	3.579	0.059	1.382	0.988–1.931
	3 = Equal pay for equal work/Temporary recruit	0.297	0.186	2.551	0.11	1.345	0.935–1.936
Average monthly income	1 = < 2000 yuan					contrast	
	2 = 2001–3000 yuan	0.359	0.211	2.907	0.088	1.432	0.948–2.165
	3 = 3001–4000 yuan	0.634	0.232	7.496	0.006	1.886	1.197–2.969
	4 = 4001–5000 yuan	0.744	0.263	7.98	0.005	2.104	1.256–3.524
	5 = >5001 yuan	0.364	0.298	1.495	0.221	1.44	0.803–2.582
The quality of sleep	1 = Very satisfied and relatively satisfied					contrast	
	2 = General	0.131	0.166	0.618	0.432	1.14	0.803–2.582
	3 = Relatively dissatisfied and very dissatisfied	0.609	0.17	12.77	< 0.001	1.838	1.317–2.567

## Discussion

### The status quo of career plateau among primary care providers

The results showed that many primary care providers had reached a career plateau state (the mean value of the overall plateau was 4.07 ± 0.77). These findings are consistent with previous findings [18], which may reflect factors such as relatively insufficient resources of basic medical and health institutions, lack of a management mechanism, and individual psychological disorders of primary care providers. The score for centralised plateau was the highest (4.48 ± 1.33), which may be related to the relatively few post settings in basic medical and health institutions [35] and the lack of opportunities to change positions at the current level. According to the guidance document issued by Jiangxi Province in China [35], there are few core management positions and a simple personnel structure in basic medical and health institutions, which makes it less likely for primary care providers to advance towards the organisation’s centre, resulting in a centralised plateau. The work content of primary care providers is also high (4.46 ± 0.85), which may be related to the absence of basic medical technology and the lack of opportunities for further study [18]. The lowest score was 2.97 ± 0.92, which may be related to the relatively stable and simple staff pyramid structure in primary care providers’ institutions. Post-hierarchical and hierarchical systems of basic medical and health institutions are reduced and the personnel structure is relatively stable, which makes it difficult for primary care providers to reach the level plateau.

### The factors associated with the career plateau of primary care providers

The results showed that marital status, weekly working hours, number of years on the job, sleep quality, and monthly average income were all associated factors for the incidence of career plateau in primary care providers.

### Marital status

The findings suggest that primary care providers with spouses faced a higher probability of facing a career plateau. This is contrary to the finding of Fu et al. [21] and may reflect the low per capita GDP in Heilongjiang Province and the inadequate salary and welfare benefits of primary care providers, who are considered medical personnel. When a primary care provider has a spouse, some of them will devote time to family life and their enthusiasm for work fades. In addition, in terms of traditional Chinese culture, primary care providers with spouses may face greater pressures of family life, such as supporting parents and raising children. Due to the distinctiveness of their work, support from the families of primary care providers may also be weakened, which may affect their ability and space for promotion to some extent.

### Working hours

The results show that the likelihood of career plateau was higher for primary care providers who worked more than 40 h a week. Working long hours also meant that primary care providers had less time to bond with family and friends and low understanding and support from them. According to the study of He et al. [4], a daily working time of more than seven hours could contribute to primary care providers’ job burnout. Simultaneously, other studies have found that the two dimensions (hierarchical and content) of the career plateau are significantly positively correlated with each dimension of job burnout, and the centralised plateau is significantly positively correlated with dehumanisation and low personal achievement [36]. However, the primary care providers who participated in this study had worked longer times and were more likely to suffer from job burnout [4], which affects work output and leads to a plateau state.

### Working life

Research shows that the number of years on the job also contributed to primary care providers experiencing a career plateau such that the more years on the job, the higher the chance of a career plateau. This is contrary to the research results of Fu et al. [21], which may reflect a lack of medical resources and access to knowledge for primary care providers in Heilongjiang Province. Regarding talent training, the organisation will train newly appointed personnel who lack sufficient work experience. Under the restrictions of institutional factors and human management abilities, the more the working years, the more confused primary care providers feel about their career prospects. Gradually, they develop a tired mentality that restricts their ability for self-improvement, reduces their chances of promotion, and makes them more likely to reach the plateau state [18].

### Quality of sleep

The results show that the likelihood of career plateau was higher for primary care providers who had the poor quality of sleep because it is directly associated with their physical health, making them nervous and anxious, generating a sense of job burnout, and affecting their daily work output, and thereby making them hit a career plateau [36]. Poor sleep quality will also trigger negative emotions towards work [37], which is not conducive to communicating with patients and is more likely to result in doctor-patient disputes, making them more likely to reach a plateau state in their occupation.

### Monthly income

The results show that the likelihood of career plateau was higher for primary care providers who earn an average monthly income of less than 5000 yuan. Most employees with an average monthly income of less than 2000 yuan are new to the job and have fewer life pressures and less burden from their families, while those with a monthly average income of 4001–5000 yuan are middle-level personnel, who face a career bottleneck and multiple pressures from family life and are prone to career plateau. Among high-level personnel whose monthly average income is more than 5000 yuan, there is limited scope for promotion, but there is also limited family pressure since most of their children have graduated, and their occupation plateau prevalence rate is thus lower than in the middle-income group.

### Improved path of career plateau in primary care providers

In terms of actual management, we should take measures to improve the career plateau status of primary care providers as follows: First, at the individual level, by encouraging and guiding primary care providers [18], planning their career path [21], and helping them communicate with others and providing feedback on a regular basis [18], they can be encouraged to establish the subjective consciousness of career development, strengthen their core qualities, and establish appropriate goals to adapt to the trend of career development without boundaries [38, 39]. Second, regarding organisational features, primary-level health service institutions should reduce the working hours of primary care providers and ensure adequate sleep time. Simultaneously, basic medical and health institutions should focus on the construction of organisational culture and provide career counselling channels and psychological counselling services for employees. This will make full use of its qualities and advantages and thus attract patient resources for basic medical and health institutions. Within the organization, a broadband salary structure should be implemented, formative evaluation and social evaluation must be incorporated into the multi-performance assessment mechanism, and a post-rotation system should be implemented [21]. The training of community-level primary care providers is conducted based on post competency, which provides additional career development opportunities for employees. For primary care providers with a long working life, the organisation should give them sufficient care, encourage them to constantly learn so as to improve themselves, provide them with necessary supportive resources, and strive to improve their psychological well-being, organisational recognition, and organisational commitment of community-level primary care providers. Third, at the social level, additional attention and support should be provided to the families of primary care providers (especially dual-career families) [40], so that their work can be better supported by their families and society. Simultaneously, various media channels should be used to reshape public perception of primary care providers so as to improve their sense of gain, happiness, and career honour [41]. Fourth, from a policy perspective, the level of remuneration and treatment for primary care providers should be improved. We should implement preferential treatment policies for talent and career promotion and provide more policy support to community-level medical and health institutions that are not qualified for scientific research. Concurrently, special legislation should be introduced to protect the personal rights and interests of primary care providers to create a good medical environment for them.

### Limitations

This current study has certain limitations. First, regarding the classification of the career plateau, researchers have divergent opinions. This study draws on the three-dimensional career plateau scale compiled by Baoguo Xie. The results may be different in other studies and require confirmation through additional research. Second, limited by time and economic cost, this research adopted a cross-sectional research method, and no follow-up survey has been carried out on primary care providers, which should be the focus of future research. Third, because of limited resources, we carried out a survey in Heilongjiang Province of China with stratified sampling, which should be further expanded in future studies.

## Conclusions

This study shows that marital status, working hours, number of years on the job, sleep quality, and average monthly income are the main factors associated with the career plateau of primary care providers. To eliminate the negative associated factors of the career plateau, we should not only provide encouragement and guidance to primary care providers, but also give them full support from the organizational, social, and policy perspectives.

Every element has both yin and yang aspects, which are in harmony with qi. While a career plateau is considered negative for employees, it also brings a period of stability. Future research should focus on the positive aspect of the career plateau, that is, what positive role a career plateau brings to employees and how to appropriately use this aspect of the career plateau to improve employees’ work output.

## Supplementary Information

**Additional file 1.** Questionnaire on career plateau prevalence and associated factors of primary health care providers. The questionnaire consists of two parts: one part is the demographic variables of primary health care providers, and the second part is the career plateau measurement scale of primary health care providers

## Data Availability

The data set used and/or analyzed in the current study may be obtained from the corresponding author upon reasonable request.

## Abbreviations

GDP: Gross Domestic Product
COVID-19: 2019 novel coronavirus disease
