# Supplementary material for: Prevalence and associated factors of the career plateau of primary care providers in Heilongjiang, China: a cross-sectional study
Source: BMC Fam Pract. 2021 Feb 17;22:38. doi: 10.1186/s12875-021-01389-w (PMC7888696; doi:10.1186/s12875-021-01389-w)
Supplement: Supplementary file 1 — Additional file 1. Questionnaire on career plateau prevalence and associated factors of primary health care providers. The questionnaire consists of two parts: one part is the demographic variables of primary health care providers, and the second part is the career plateau measurement scale of primary health care providers [file 12875_2021_1389_MOESM1_ESM.docx]

**The career plateau related questionnaires for primary health care providers**

Investigator’s self-introduction and illustration:

This survey is about to inspect the career plateau of primary health care providers.

Primary health care providers are playing an important role in the construction of medical and health system in our country. They are also the guard of people health. Through these questionnaires, we hope to fully realize that your feelings and tastes for current job. After that, we can make the valuable advice to perfect the primary health services. We will respect your privacy strictly. The collected information will only be used in academic analysis, and will never be imparted for any departments or institutions. Please write these questionnaires according to your realities. Thanks a lot for your cooperation. We wish you smooth work and health.

Basic information

1. Gender: ①male ②female;
2. Marriage status: ①have a spouse ②no spouse;
3. Ages: ①less than or equal to 30 ②31-40 ③41-50 ④Greater than or equal to 50;
4. Record of formal schooling: ①high school degree or below ②university college ③ university degree or above;
5. Working fixed number of year: ①no more than 10 years ②11-20 years ③ more than 20 years;
6. Average monthly income：①2000 yuan of the following ②2000-3000 yuan ③3001-4000 yuan ④4001-5000 yuan ⑤more than 5000 yuan;
7. Weekly working hours: ①less than or equal to 40 hours ②more than 40n hours;
8. Flexible job system: ①yes ②no
9. Type of personnel post allocation: ①utilities staffing ②appointment system ③equal pay for equal work /temporary recruit
10. Assessment for sleep quality: ①very satisfied or quite satisfied ②just so so ③ very dissatisfied or quite dissatisfied

Career plateau

1. **Content plateau**
2. My current job can enrich my working skills.

①totally agreed ②quite agreed ③a bit agreed ④a bit disagreed ⑤quite disagreed ⑥totally disagreed

1. I can learn something new from my current job.

①totally agreed ②quite agreed ③a bit agreed ④a bit disagreed ⑤quite disagreed ⑥totally disagreed

1. I usually contact something new that is related to my job.

①totally agreed ②quite agreed ③a bit agreed ④a bit disagreed ⑤quite disagreed ⑥totally disagreed

1. My current job can broaden my horizons.

①totally agreed ②quite agreed ③a bit agreed ④a bit disagreed ⑤quite disagreed ⑥totally disagreed

1. My current job requires me to learn new knowledge constantly.

①totally agreed ②quite agreed ③a bit agreed ④a bit disagreed ⑤quite disagreed ⑥totally disagreed

1. My job missions and activities have already become the repetitive tasks.

①totally agreed ②quite agreed ③a bit agreed ④a bit disagreed ⑤quite disagreed ⑥totally disagreed

1. **Center plateau**
2. I get more power from superiors.

①totally agreed ②quite agreed ③a bit agreed ④a bit disagreed ⑤quite disagreed ⑥totally disagreed

1. Superiors usually require me to be responsible for some important transactions.

①totally agreed ②quite agreed ③a bit agreed ④a bit disagreed ⑤quite disagreed ⑥totally disagreed

1. I am usually responsible for some important transactions in my work unit.

①totally agreed ②quite agreed ③a bit agreed ④a bit disagreed ⑤quite disagreed ⑥totally disagreed

1. In mu current job, I have more right to speak.

①totally agreed ②quite agreed ③a bit agreed ④a bit disagreed ⑤quite disagreed ⑥totally disagreed

1. I can get more organizational resources from my work unit.

①totally agreed ②quite agreed ③a bit agreed ④a bit disagreed ⑤quite disagreed ⑥totally disagreed

1. I get the chance to engage in the decision making in my work unit.

①totally agreed ②quite agreed ③a bit agreed ④a bit disagreed ⑤quite disagreed ⑥totally disagreed

1. **Hierarchy plateau**
2. I get limited space to be promoted in my work unit.

①totally agreed ②quite agreed ③a bit agreed ④a bit disagreed ⑤quite disagreed ⑥totally disagreed

1. I can never get a higher position in my work unit.

①totally agreed ②quite agreed ③a bit agreed ④a bit disagreed ⑤quite disagreed ⑥totally disagreed

1. The possibility of get promoted is little in my work unit.

①totally agreed ②quite agreed ③a bit agreed ④a bit disagreed ⑤quite disagreed ⑥totally disagreed

1. I still can get promoted from superiors.

①totally agreed ②quite agreed ③a bit agreed ④a bit disagreed ⑤quite disagreed ⑥totally disagreed

**REFERENCES**

Xie B-G, Long L-R, Zhao Y-J. Development of Career Plateau Questionnaire and Research on Its Validity and Reliability. Chinese Journal of Clinical Psychology.2008;16:344-7.DOI:10.16128/j.cnki.1005-3611.2008.04.008
